# On the Importance of Field Studies for Testing Theory-Driven Behavioral Change Interventions in (Sustainable) Tourism

**DOI:** 10.1177/00472875241253009

**Published:** 2024-05-31

**Authors:** Emil Juvan, Oscar Yuheng Zhu, Bettina Grün, Sara Dolnicar

**Affiliations:** 1 Faculty of Tourism Studies Turistica, University of Primorska, Portorož, Slovenia; 2 UQ Business School, The University of Queensland, Brisbane, QLD, Australia; 3 Institute for Statistics and Mathematics, Vienna University of Economics and Business, Vienna, Austria

**Keywords:** sustainable tourism, food waste, plate waste, field experiment, survey experiment

## Abstract

Practical measures to entice tourists to behave in environmentally sustainable ways are urgently needed. The effectiveness of such measures is typically tested in survey experiments. This study demonstrates that this approach can be misleading. We test two messages aimed at reducing buffet food waste. One builds on established theories of human behavior (theory of planned behavior, value-belief-norm theory); it assumes that changing beliefs by providing information triggers behavior change. The second message builds on hedonic psychology; it attempts to change behavior through humor, presenting the pro-environmental behavior as enjoyable. In the survey experiment, the belief-based message significantly increases intentions to reduce plate waste; but both messages fail to change behavior in a real hotel. These insights have methodological and practical implications: the effectiveness of new practical measures developed to trigger specific tourist behaviors must be tested in the field before reliable managerial recommendations can be derived.

## Introduction

The world is “*on a pathway to global warming of more than double the 1.5-degree limit*” (United Nations, 2022a). Although the Paris Agreement has called for a 45% emission reduction by 2030, progress toward this goal is insufficient (United Nations, 2015a, 2022b). In contrast to many other sectors, tourism depends on natural resources, which represent a key driver of tourist visitation (e.g., Garms et al., 2017; Kim, 1998). Yet, tourism also harms these very resources it existentially depends upon by using extensive amounts of fresh water (Gössling et al., 2012; Rico et al., 2020), emitting 8% of all global CO_2_ emissions, generating substantial amounts of solid waste (Diaz-Farina et al., 2020) and food waste (De Visser-Amundson, 2022; Gössling et al., 2011) and by discharging toxic sludge (Moscovici, 2017).

Reducing travel activity is not a desirable solution because: tourism is a vital income source for many host communities and nations (Uysal et al., 2016); going on vacation increases people’s life satisfaction (Dolnicar et al., 2012); and many people view travel as an integral part of their identity (White & White, 2004) and lifestyle choice (S. A. Cohen et al., 2015). The focus, therefore, must shift toward making the tourism sector more environmentally sustainable. One way of achieving this is by enticing tourists to behave in more environmentally sustainable ways—an approach highlighted by the Intergovernmental Panel on Climate Change, which states that “*changes to behavior can result in a 40-70% reduction in greenhouse gas emissions by 2050*” (IPCC, 2022). Behavioral change interventions represent a key strategy for climate change mitigation across all industry sectors, including tourism.

Previous research has shown that, although 60% of tourists state they consider the environmental impact of their trip (Karlsson & Dolnicar, 2016), most fail to convert their pro-environmental attitudes to sustainable behavior when they go on vacation (Juvan & Dolnicar, 2014). The attitude-behavior gap has been identified in relation to many different environmentally sustainable behaviors, including choosing eco-certified tour providers (Karlsson & Dolnicar, 2016), purchasing green apparel (Wiederhold & Martinez, 2018), and ordering sustainable meals (Yamoah & Acquaye, 2019). This attitude-behavior gap can impact the validity of self-report survey measures used in studies of many different behaviors. This study aims to address this challenge by directly comparing the validity of results from a survey and a field experiment using as the context a specific environmentally friendly behavior: the generation of food waste at all-you-can-eat buffets (Juvan et al., 2018). This is an important area because tourists perceive vacations as special occasions and give themselves permission to eat more (Gössling & Peeters, 2015) and waste more food (Liu et al., 2022). In addition, food waste has both environmental and economic implications. Food waste contributes to global warming, with 8% to 10% of global greenhouse gas emissions resulting from food that is ultimately discarded (United Nations Environment Programme, 2021). For businesses, the economic loss caused by food waste is also considerable (Waste and Resources Action Programme, 2020); it adds unnecessary cost for food purchase and disposal. Despite the United Nations (2015b) target to halve food waste by 2030, food waste is not decreasing (EUROSTAT, 2023). Previous research has classified food waste into various stages, such as production, transportation, and consumption (K. Fisher et al., 2013). Edible food left behind uneaten by consumers—referred to as plate waste—has more significant environmental and economic consequences because all emissions of the food production cycle are wasted and additional emissions are generated when food decomposes in landfill (K. Fisher et al., 2013). This is why the present study uses plate waste as the context for what is primarily methodological study, which compares the validity of results from a survey experiment with those of a field study to determine if newly developed behavior change interventions are effective in reducing plate waste.

While our study focuses on food waste as the research context, the results from the comparison we present can be extrapolated to other environmentally sustainable behaviors that also exhibit an attitude-behavior gap. Specifically, this study investigates whether survey experiments are sufficient to derive valid conclusions about the effectiveness of behavioral change interventions, or whether field studies are necessary. This is a key methodological insight, which is critically important to guide future experimental studies testing the effectiveness of behavioral change interventions that have the potential of making material contribution to the environmental sustainability of the tourism sector. If survey experiments generate misleading results, tourism managers risk adopting behavior change interventions—for any type of tourist behavior—that risk failing when deployed in real-world contexts.

## Literature Review

### Methodological Approaches to Testing the Effectiveness of Behavior Change Interventions

Self-report surveys and survey-based experiments are commonly used to assess the effectiveness of behavior change interventions in tourism (Dolnicar, Zinn & Demeter, 2023; Viglia & Dolnicar, 2020). Surveys represent a low-cost and time-efficient approach to accessing information from a large sample. Some behaviors—such as past pro-environmental behaviors on vacation and behavioral intentions—can be measured only in self-report studies. A well-designed survey is also able to measure whether a behavior change intervention triggers the corresponding psychological constructs postulated to induce behavior change (Sparks et al., 2013). As a result, survey studies are extensively used in tourism research to assess the effectiveness of behavior change interventions, including promoting sustainable transport choices (Brög et al., 2009), inducing responsible water and electricity use (Tussyadiah & Miller, 2019) and adopting post-visit environmentally responsible behaviors (Ballantyne & Packer, 2011). Responses from survey studies, however, are prone to biases, including social desirability bias (R. J. Fisher, 1993), which occurs when respondents answer questions in a way that paints a favorable picture of them, rather than reflecting the truth (Paulhus, 1984). Social desirability bias can inflate self-reported environmentally sustainable tourist behavior by 74% (Juvan & Dolnicar, 2016). Additionally, item-level test-retest reliability can be very low (Dolnicar et al., 2022). When using a seven-point bipolar answer format, for example, respondents only provide the same response twice in the absence of external changes in 47% of cases (Dolnicar, 2021). Importantly, real behavior (specifically socially desirable behavior, such as pro-social or pro-environmental behavior) cannot reliably be measured in surveys because stated behavioral intentions are particularly prone to social desirability bias (Juvan & Dolnicar, 2017). Another possible reason for the gap between stated behavioral intentions and actual observed behavior is the diverse nature of the external factors (Thaler & Sunstein, 2009) affecting real behavior. Tourists themselves report 19 different reasons for the gap between people’s intentions to behave in environmentally sustainable ways and their actions (Juvan & Dolnicar, 2021). In the context of plate waste behavior, they provide 12 different reasons (Dolnicar & Juvan, 2019). The diversity of these internal and external factors is difficult to capture in survey experiments.

An alternative way of testing the effectiveness of behavior change interventions is to run laboratory or field experiments (Harrison & List, 2004). Laboratory experiments observe specific types of behaviors in a controlled environment (Gneezy, 2017; Viglia & Dolnicar, 2020). Laboratory experiments are less prone to social desirability bias, but the observed behavior is rarely the main behavior of interest to the researcher. For example, attention paid to carbon offsetting appeals when booking a flight (Babakhani et al., 2017) or ordering a burger (Babakhani et al., 2020) can validly be measured with fixations in eye tracking studies. But attention as captured by eye fixations does not directly translate to actual consumer purchase decisions. As such, laboratory experiments offer additional insights to survey experiments, but still fall short of being able to ascertain if new behavior change intervention influence tourist behavior.

Field experiments can overcome this limitation by testing behavior change interventions in real-life contexts (Gerber & Green, 2012), thus ensuring high external validity (Viglia & Dolnicar, 2020). True field experiments are almost impossible to conduct in real-life tourism contexts because they require random assignments of study participants to experimental conditions. In a hotel, for example, this would imply being able to control which hotel guests stay during the control period and which during the experimental period. Because this is not possible, field studies in tourism are typically quasi-experimental field studies; they have all features of a field experiment except random assignment of study participants. Even quasi-experimental field studies remain rare in tourism research because they are costly and time-consuming. A recent review of field studies aimed specifically at improving the sustainability of tourism, concludes that only few behaviors and interventions have been extensively tested in the field (Demeter et al., 2023). Towel reuse and food waste reduction have received most attention. In terms of theoretical constructs, beliefs and social norms are the two most used leverage mechanisms; this is despite the relatively low success rate of these approaches (58% and 47%, respectively; Demeter et al., 2023).

### Successful Behavior Change Interventions for Sustainable Tourism

The study of sustainable tourism has a long and proud history (Bramwell & Lane, 1993). Yet surprisingly few tangible measures have been proven to reduce the environmental footprint of tourism, with the main focus on reducing water use (Tiefenbeck et al., 2019), plate waste (Dolnicar et al., 2020; Kallbekken & Sælen, 2013), and routine hotel room cleaning (Dolnicar et al., 2019a; Kneževič Cvelbar et al., 2021); directing online bookings to low-carbon options (Araña & León, 2016); encouraging carbon offsetting; using recycled paper serviettes (Dolnicar et al., 2019b), and increasing towel reuse (Baca-Motes et al., 2013; Goldstein et al., 2008; Gössling et al., 2019).

These field experiments allow statements about whether specific practical measures change real behavior. Yet, field experiments are uncommon, presumably because of the cost and effort associated with conducting them. A recent review of food waste reduction studies shows that only 42% (123 out of 292) of them quantified food waste generation or reduction in any way (Reynolds et al., 2019). Of those 123 studies, nearly one third relied on consumer self-reports (Reynolds et al., 2019). Because self-reports are prone to social desirability bias (R. J. Fisher, 1993) and respondents tend to overreport socially desirable behavior (Krumpal, 2013), the external validity of survey experiments is reduced, limiting the generalizability of findings to real-world settings (Viglia & Dolnicar, 2020). In the field of environmental psychology, social desirability bias is regarded as a major factor decreasing the validity of the result (Vesely & Klöckner, 2020). Yet, self-reported behavior is still commonly used as a key dependent variable in tourism studies (e.g., Miller et al., 2015; Tan & Wu, 2016; Wu et al., 2021). To assess whether survey experiments alone are indeed insufficient to learn about behavior change in the real world, a comparison of outcomes from survey and field experiments is required. In this study, we offer such a methodological comparison.

## Methodology

We developed and tested two alternative interventions (table signs) aimed at reducing buffet plate waste in a five-star hotel. We use plate waste generation behavior in this study but do not expect results to be substantially different for other socially desirable environmentally sustainable behaviors. We conducted a quasi-experimental field study to test if the interventions changed real behavior (as opposed to stated behavioral intentions in a survey study), specifically a reduction in plate waste generated by tourists at a hotel buffet. We used a survey experiment (1) to derive the self-reported behavioral intentions, (2) to ensure that the interventions did not lead to negative emotional reactions, and (3) to determine if the psychological mechanisms assumed to be triggered by the interventions are successfully triggered. The latter served as a manipulation check. Manipulation checks provide insights into changes in latent psychological constructs that are not observable (Sparks et al., 2013; Viglia & Dolnicar, 2020).

**Figure 1.**
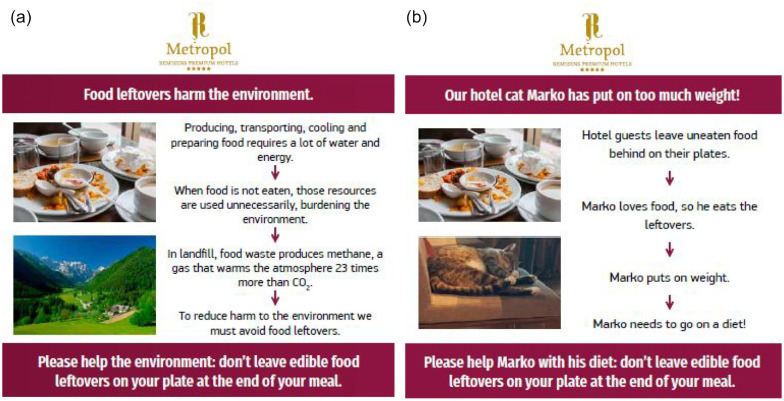
Table signs used in the experimental conditions: (a) environmental beliefs and (b) humor.

While both table signs were designed in line with the hotel branding, the messages contained on the two table signs were informed by different theories. The first intervention used the theory of planned behavior (Ajzen, 1991) and value-belief-norm-theory (Stern, 2000) as a basis and, in line with these theories, attempted to leverage environmental beliefs to achieve behavior change by informing guests about how food leftovers negatively affect the environment and concluded with: “*Please help the environment: don’t leave edible food leftovers on your plate at the end of your meal*” (see Figure 1a). We designed this intervention with the specific aim of activating beliefs postulated by both the theory of planned behavior and the value-belief-norm theory. In the message, we first inform people that food waste adversely impacts the environment across production, transportation, and decomposition stages. This information is intended to trigger the construct of awareness of consequences (Stern, 2000). In the second part of the message, we communicate to people that their behavior can reduce food waste and, in so doing, minimize environmental harm. This information is designed to trigger the construct of ascription of responsibility (Stern, 2000). The belief-based intervention included 77 words (403 characters) and two photos.

The second table sign leveraged the effect of humor. Humor has been effective in triggering behavior change in other contexts (Cernerud & Olsson, 2004) and aligns well with tourism, which is an inherently hedonic activity (Pearce, 2009). Humor can help people process information actively and enhance concentration in a hedonic context such as tourism (Kahneman et al., 1999; Pearce, 2009). This is because humor is particularly promising in “*tension filled situations*” (Pabel & Pearce, 2016, p. 192) and situations where beliefs and behavior do not align (Martin, 2007), as is the case with environmental sustainability and the pursuit of pleasure. Tourism businesses are increasingly adopting humor as a strategy (Johnson & Ball, 2000; Shaw et al., 2010). Tourists acknowledge humor and use humor to cope with unpleasant situations, such as overcrowded places or embarrassing situations (Pabel & Pearce, 2015). Humor enhances “*comfort, concentration and connection levels*” (Pabel & Pearce, 2016, p. 190). Using humor in guided tours increases tourist engagement in desired behaviors (Zhang & Pearce, 2016). Evidence from previous tourism research shows that humor is often regarded as an important strategy for tourism businesses (Johnson & Ball, 2000; Shaw et al., 2010) because it is particularly effective in triggering positive emotions (Pabel & Pearce, 2015), which, in turn increases the effectiveness of pro-environmental behavioral interventions (Schneider et al., 2021). A humor-based behavioral intervention, therefore, “*can be leveraged to promote desirable behavior change*” (Shiota et al., 2021, p. 222). For these reasons, leveraging the psychological construct of humor may have potential to induce behavior change in the vacation context.

We implemented the humor intervention by informing guests that food leftovers negatively affect the health of the already overweight hotel cat Marko and concluded with: “*Please help Marko with his diet: don’t leave edible food leftovers on your plate at the end of your meal*” (see Figure 1b). We designed the humor-based intervention with the help of hotel staff who reported that Marko is a “regular visitor” to the restaurant, that guests often pat the cat and offer Marko food leftovers. For that reason, hotel staff argued, tourists are likely to react with amusement to a message using Marko to motivate responsible eating behavior. We presented the intervention to six restaurant staff members, and 17 individuals randomly intercepted at the walking promenade of the city where hotel is located. The message with a photo of Marco was printed on white paper of the same size as the intervention material and presented to individuals with the question *“Imagine seeing such a photo and message on a table while having breakfast and/or dinner in a hotel where you stay; how would this make you feel*?” Feedback from this manipulation check confirmed that the intervention was appealing, that it was not perceived as annoying, and that it indeed triggered positive emotions, including feeling amused and entertained.

The humor intervention included 55 words (247 characters) and 2 photos. This intervention was shorter because it only had to communicate the humor. In contrast, the belief-based message had to communicate two constructs: awareness of consequences and ascription of responsibility (Stern, 2000). There is a risk that the longer message could either reduce or increase the effectiveness of the intervention (Cole et al., 1997).

### Study 1: Survey Experiment and Manipulation Check

We recruited 317 respondents via the online recruitment platform Prolific Scholar because online surveys are less prone to capturing social desirability bias than face-to-face survey method (Berzelak & Vehovar, 2018). We did not disclose the purpose of the study as an additional measure of preventing social desirability bias (Juvan & Dolnicar, 2016; Krosnick, 1999). Only respondents who have stayed at a hotel and have eaten at a hotel buffet in the past 5 years qualified for the survey. We implemented these criteria to ensure that we recruited a similar market segment to guests in the field experiment.

All survey respondents were primed to imagine being on a relaxing beach holiday where an all-you-can-eat dinner buffet is included in the hotel price. We used images and stimuli that captured an atmosphere similar to that at the hotel where we conducted the field experiment. We informed respondents that plate waste is a common problem at buffets and that the average guest leaves about 100 g of uneaten food behind on their plate after each meal. We then asked respondents to imagine going to the buffet dinner. We randomly assigned respondents to one of three conditions. The control group (*n* = 110) did not see a sign. Members of experimental group 1 (*n* = 102) saw the sign targeting environmental beliefs. Members of experimental group 2 (*n* = 105) saw the sign with Marko, the cat. After seeing the signs, respondents answered the following question: “*Which percentage of the food you take from the buffet would you leave behind uneaten? Please remember that the average guest leaves 100g of uneaten food behind at each meal.*” Respondents recorded their answers using a slider scale ranging from 0% to 100%.

We tested whether the interventions activated the intended emotions by asking: “*Now please remember the sign you saw. How did it make you feel?*”. Respondents chose “Yes” or “No” for each of the following 10 emotions presented to them: *wanting to eat up everything on my plate*, *interested*, *responsible*, *concerned*, *sustainable*, *guilty*, *amused*, *entertained*, *upset*, and *annoyed*. Based on value-belief-norm theory (Stern, 2000) and existing empirical evidence about the attitude–behavior gap in sustainable tourism (e.g., Juvan & Dolnicar, 2014), we expect both interventions to make respondents feel *guilty, concerned, responsible, sustainable, upset*, and *wanting to eat up everything on their plate*, while after seeing the sign with the cat respondents will feel, more *entertained, amused, interested*, and less *annoyed*.

We summarize stated behavioral intentions for each experimental condition using descriptive statistics (mean, standard deviation, skewness, excess kurtosis, median, and quantiles) to assess differences in location as well as shape. A shift in location is tested for all three conditions using a Kruskal-Wallis rank sum test as well as a Wilcoxon rank-sum test for the comparison between control and each of the experimental conditions. The power of the Wilcoxon rank sum test for a sample size of 100 in each group and a significance level of 5% is assessed using sampling-based methods. Observations obtained in the control condition are bootstrapped using 10,000 replications with different positive shifts being added for the treatment condition while also taking the admissible range of values into account. We analyze activated emotions by visualizing agreement percentages for each experimental condition across the 10 emotions using bar plots. We then test for differences in proportions using a chi-squared test for each emotion. The power of the chi-squared test is investigated for a sample size of 100 in each group and a significance level of 5% based on the normal approximation for the binomial distribution using different success probabilities for the two experimental conditions.

### Study 2: Quasi-Experimental Field Study

We conducted a field study to ensure we could draw causal conclusions about actual tourist behavior that are valid for the hotel context (Viglia & Dolnicar, 2020). Our study is a quasi-experimental field study because it is not possible to randomly assign tourists to a specific experimental condition when conducting the experiment during normal hotel business operations. The field site was a five-star hotel at a popular Slovenian seaside tourist destination.

The dependent variable was average plate waste generated at the breakfast and dinner buffets per person per day in grams. Plate waste was measured using a fully automated system that monitors plate waste (Dolnicar, Gray et al., 2023). This system uses a floor scale that was placed under the plastic bins used in the kitchen to dispose of the plate waste and upload the plate waste data automatically (Dolnicar, Gray et al., 2023). These bins are not used for kitchen preparation waste. The bins include food and non-edible biodegradable items such as serviettes. The number of people who ate breakfast and dinner on any given day was provided by the hotel and collected by a staff member located at the entrance to the dining area. This staff member welcomes guests and notes their room numbers. In addition, we obtained de-identified daily guest mix data from the hotel. We had an equal distribution of male (48.6%, *n* = 4,219) and female (51.4%, *n* = 4,461) guests during the field experiment with an average stay duration of about four days.

We measured plate waste per person per meal for the control condition and the two experimental conditions. Both the control and the experimental conditions ran during the peak summer tourist season. The control condition ran from 1st to 21st of July 2019, and effectively represented the status quo of operations at the hotel. The first experimental condition was implemented between the 24th of July 2019 and the 11th of August 2019 and involved an information intervention providing facts about the negative environmental impacts of food waste (see Figure 1a). The second experimental condition—fielded between 12th of August 2019 and 2nd of September 2019—involved the humor message intervention (see Figure 1b).

The messages were presented as a cube-shaped table sign where each side contained the same information as shown in Figure 1 in a different language, covering the main languages used by guests in this hotel (Slovenian, English, German, and Italian). The design of the table signs was in line with the hotel branding.

To check whether the signs were noticed and triggered the intended emotions—make people giggle and increase knowledge about wastefulness and the environmental impacts of plate waste—we conducted a voluntary survey study, asking people at checkout to answer a few questions about their dining experience. Because this was a voluntary survey, the response rate was low: 22 tourists in the control condition completed the survey, 32 in the belief condition, and 24 in the humor condition. In both experimental conditions respondents indicated that they have noticed the table sign more often than tourists in the control group (control group: 32%, experimental group 1—environmental beliefs: 69%, experimental group 2—humor: 92%; Fisher’s exact test: *p* < .001), with a slight indication that the table sign might be noticed more frequently in the humor condition (Fisher’s exact test: *p* = .05). When inspecting which emotions were triggered by the table signs, 68% of respondents in the humor condition indicated that they were amused by the table sign, but only 14% did so in the belief condition (Fisher’s exact test: *p* = .001). In the belief condition 73% of respondents agreed to feel sustainable, while only 46% of respondents agreed to this statement in the humor condition, but this difference was not statistically significant (Fisher’s exact test: *p* = .12).

For each day and meal, we calculated the average plate waste per person in grams. Additional information available for each of these daily meal-specific observations consists of the number of guests, the percentage of guests having this meal for the first time at the hotel, the percentage of guests having this meal for the last time at the hotel, the percentage of guests being male, the percentage of guests with different countries of origin (categorized into Austria, Italy, Germany, Hungary, Slovenia, the remaining Eastern European and Balkan countries and all other countries of origin) and from different age groups (categorized into the following age groups in years: 0–6, 7–14, 15–24, 25–60, and 61–100).

The additional information is summarized separately for each meal and experimental condition using the empirical mean, standard deviation, skewness, and excess kurtosis. We assess differences in the number of guests across experimental conditions for each meal using *F*-tests comparing linear regression models controlling for weekday effects with and without experimental condition as independent variable. We assess differences in guest characteristics across experimental conditions for each meal using likelihood ratio tests. We compare logistic regression models controlling for weekday effects, both with and without the experimental condition as an independent variable. A time series plot indicating the experimental conditions using color-coding visualizes the average plate waste per person (in g) for each meal. To assess differences in plate waste across experimental conditions, weighted linear regression models are fitted using the average plate waster per person (in g) as dependent variable, the number of guests as weights, the guest characteristics as control variables and the experimental condition as independent variable. The effect of the experimental condition results from comparing the model with and without the experimental condition as independent variable using an *F*-test and by determining the mean and the 95% confidence intervals for these effect estimates. Based on the daily control measurements of plate waste per person for breakfast and dinner, a power analysis is performed for a two-sample *t*-test using the observed standard deviation as well as different shifts in mean values for a sample size of 20 and 30 in each group. Note that this sample size corresponds to the number of daily average plate waste values per meal and person. These averages are based on several hundred people attending each meal every day and, assuming independent and identically distributed plate waste values for each individual per meal, the effective sample size behind these mean values would correspond to the sum over the daily number of persons for this meal. The study received approval from the university human ethics committee (2019/HE001609; 2022/HE001344).

### Impact of COVID-19 on Our Research

Our primary data (field experiment) was collected before COVID-19. However, the implications and conclusions of our study are not affected by the pandemic, because it examines fundamental mechanisms driving human behavior. Specifically, we study how two alternative behavior change interventions—two table signs containing messages based on different theories of human behavior—affect food waste generation at a hotel buffet. Although some food outlets changed their serving style during the pandemic, which may have altered the food waste generated (Chang, 2022), evidence suggests that the tourism industry has largely focused on re-establishing the pre-pandemic status after the COVID-19 disruption because the dominant role of relational and normative expectations (Zhu & Dolnicar, 2022). This is exactly what happened at the hotel where the study was conducted; as soon as hotels re-opened and buffets were again permitted, buffet food service resumed. There is no obvious reason, therefore, to believe that our results are biased by the timing of data collection.

## Results and Recommendations

### Study 1: Survey Experiment and Manipulation Check

Table 1 summarizes the stated behavioral intentions across all three experimental conditions. The location measures (mean and median) indicate that stated food waste is highest for the control condition, followed by the humor and the belief conditions. The quantiles also indicate that the values are highest for the control condition with the belief condition outperforming the humor condition, particularly for the extreme 10% and 90% quantiles.

**Table 1.** Summary of the Behavioral Intentions for the Three Experimental Conditions Using Number of Observations (*N*), Empirical Mean (Mean), Standard Deviation (*SD*), Skewness (Skew.), and Excess Kurtosis (Kurt.) as Well as the Median and Several Other α-quantiles Q_α_.

Condition	*N*	Mean	*SD*	Skew.	Kurt.	Q_0.10_	Q_0.25_	Median	Q_0.75_	Q_0.90_
Control condition	110	24.2	20.9	1.4	1.7	5.0	10.0	20.0	34.5	50.5
Environmental beliefs condition	102	17.6	19.2	1.6	2.6	0.0	5.0	10.0	25.0	35.0
Humor condition	105	21.2	20.4	1.9	3.8	2.4	5.0	16.0	30.0	50.0

The differences in median values are significant according to a Kruskal-Wallis rank sum test (χ^2^ = 8.31, *df* = 2, *p* = .02), with the belief condition having a significantly lower median value than the control condition (*W* = 6,885; *p* = .004) whereas the difference in median values is not significant at the 5% level for humor versus control condition (*W* = 6,377; *p* = .186). The power analysis indicates that a sample size of *n* = 100 would not have sufficient power to identify a median shift of five (power = 0.72), while the power is already sufficiently high for a median shift of six (power = 0.97).

**Figure 2.**
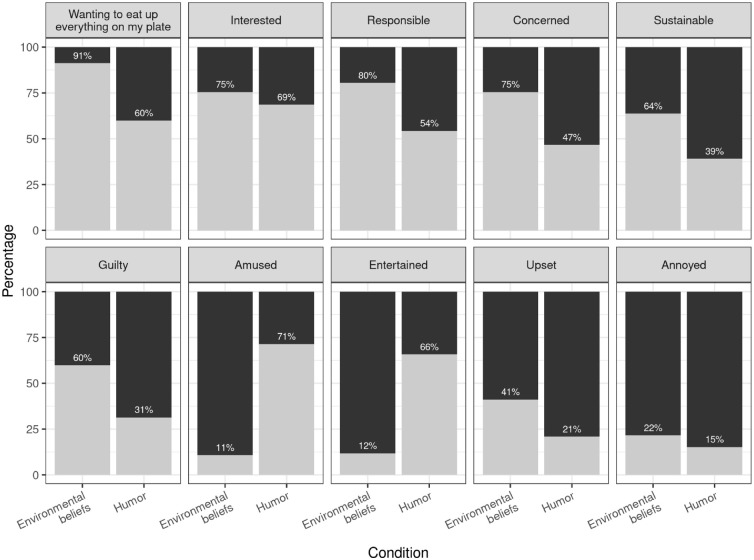
Percentages of agreement with the 10 emotions being evoked by the signs of the two experimental conditions.

Figure 2 visualizes the results of the manipulation check assessing if the signs trigger the intended emotions. The plot contains the percentages of agreement for the respondents in the two experimental conditions for each of the 10 emotions shown separately in panels with the panel label indicating the emotion. The emotions are ordered by overall agreement percentages across both experimental conditions.

The highest associated emotion overall is “*Wanting to eat up everything on my plate.*” This emotion is triggered significantly more by the belief condition with 91% compared to the humor condition with 60% (chi-squared test: χ^2^ = 25.4, *df* = 1, *p* < .001). In both conditions, respondents indicate that they feel interested (75% and 69%), with the difference insignificant at the 5% level (χ^2^ = 0.9, *df* = 1, *p* = .34). A clear majority in the belief condition also feels *responsible*, *concerned*, *sustainable*, and *guilty*, while the levels of agreement with these emotions are significantly lower for the humor condition (all *p* < .001). In contract, the humor message triggers the emotions *amused* and *entertained* (all *p* < .001), showing that the message had the intended effect. Only few respondents express being *upset* or *annoyed* with the former being slightly higher for the belief condition (*p* = .003). Levels of annoyance are not significantly different (*p* = .32). The power analysis indicates that a sample size of *n* = 100 in each group would not have sufficient power to identify a difference in proportion of 0.1 in case the proportions themselves vary between 0.1 and 0.9 (power between 0.29 and 0.51), while the power is already sufficiently high for a difference in proportion of 0.2 in case the proportions themselves vary between 0.1 and 0.9 (power between 0.81 and 0.94).

The results from this survey experiment suggest that the message targeting environmental beliefs has the potential to significantly reduce plate waste at hotel buffets. In addition, there is very little risk of any of the two messages upsetting or annoying hotel guests. Based on these findings, researchers would recommend that hotels operating buffet meal services should deploy the environmental belief-based table sign to prevent unnecessary food waste. The humor message, while not upsetting guests, does not show promise in the survey experiment.

### Study 2: Quasi-Experimental Field Study

Table 2 provides an overview of the plate waste data collected during the field study. For each meal and experimental condition, the table contains the number of daily observations as well as the empirical mean and standard deviation of the daily plate waste per person per day (in g) and the number of guests. The power analysis based on the control measurements indicates that for a relative decrease of 10% in average waste per person and day the power is insufficient for the sample size of *n* = 20 (power = 0.54 for breakfast and 0.58 for dinner), and *n* = 30 (power = 0.72 for breakfast and 0.76 for dinner). By contrast a relative decrease of 15% in average waste per person and day leads to a power above 0.8 for both sample sizes (*n* = 20: power = 0.87 for breakfast and 0.90 for dinner; *n* = 30: power = 0.97 for breakfast as well as dinner).

**Table 2.** Summary of Plate Waste per Person and Day (in g) and Number of Guests for Each Meal and Experimental Condition Based on the Number of Observations (*N*), the Empirical Mean (Mean), the Standard Deviation (*SD*), Skewness (Skew.), and Excess Kurtosis (Kurt.).

Meal	Condition	*N*	Waste per person and day (in g)				Number of guests			
			Mean	*SD*	Skew.	Kurt.	Mean	*SD*	Skew.	Kurt.
Breakfast	Control	22	42.2	6.3	−0.2	−1.3	482.6	57.4	−1.0	0.4
	Environmental beliefs	19	42.5	4.5	0.4	−1.0	537.8	32.1	−1.3	2.0
	Humor	22	41.1	7.1	0.6	−0.2	483.2	57.1	−1.4	2.3
Dinner	Control	22	99.2	14.1	0.1	−1.1	355.8	33.6	0.2	−1.1
	Environmental beliefs	18	102.6	17.8	0.1	−0.8	399.7	27.2	−0.1	−0.8
	Humor	20	100.9	12.3	0.6	−0.3	351.9	56.7	−1.4	1.4

**Figure 3.**
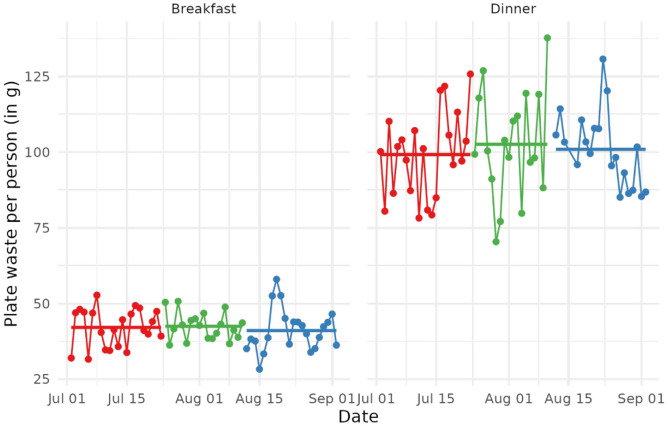
Plate waste pattern for breakfast and dinner. (Red—control condition, green—environmental beliefs condition, and blue—humor condition).

Complementing Table 2, Figure 3 visualizes the average plate waste per person in grams per day and meal for the three experimental conditions, separately for breakfast and dinner. The daily average plate waste is indicated by a bullet and these bullets are joined by lines between consecutive days and within each experimental condition. Colors indicate the different experimental conditions: control (red), environmental beliefs (green), and humor (blue). In addition, the overall average plate waste per person (in g) per day and meal for each experimental condition is given by straight lines.

A few key findings can be derived from Table 2 and Figure 3. First, the average plate waste is much lower for breakfast than dinner. Second, plate waste fluctuation across days is substantially higher for dinner. This is likely due to the breakfast buffet containing the same set of food items every day (across all experimental conditions). In contrast, at the dinner buffet, the menu changes daily, thus also increasing the likelihood of people taking something they do not enjoy or overserve themselves due to their eyes being bigger than their bellies (Dolnicar & Juvan, 2019) and, as a result, leave behind uneaten food. Third, and most importantly for the present study: neither of the two experimental conditions significantly reduces the amount of plate waste generated (as opposed to the control group). Based on these findings, a behavioral scientist could not recommend deploying any of the signs at a hotel. This stands in direct contract to the recommendation they would make had the conducted a survey experiment only.

Guest characteristics differ considerably across the experimental conditions as well as the two meal types—Table 3 shows the empirical mean and standard deviation for the daily number of guests and the guest characteristics for each meal type and experimental condition. These results indicate that accounting for differences in guest characteristics is warranted when estimating the effects of the experimental conditions. The guest characteristics are included as control variables in the regression models estimating the effects of the experimental conditions on the average plate waste.

**Table 3.** Summary of Number of Guests and Demographic and Guest Characteristics for Each Meal and Experimental Condition Based on the Empirical Mean and the Standard Deviation (in Round Parentheses).

Meal	Variable	Control group	Environmental beliefs	Humor	*p*-value
Breakfast	Number of guests	482.6 (57.4) [−1.0; 0.4]	537.8 (32.1) [−1.3; 2.0]	483.2 (57.1) [−1.4; 2.3]	.002
	First day (%)	27.9 (6.6)	23.8 (6.4)	25.5 (7.2)	<.001
	Last day (%)	24.9 (9.3)	24.7 (7.2)	26.6 (10.1)	<.001
	Male (%)	47.4 (1.6)	47.8 (1.2)	49.2 (2.5)	.020
	Country of origin (%)				
	Austria	17.8 (3.0)	19.4 (3.2)	19.4 (5.3)	.007
	Italy	11.0 (4.9)	10.8 (4.4)	27.2 (7.9)	<.001
	Germany	12.1 (2.5)	14.5 (2.6)	10.6 (3.3)	<.001
	Hungary	11.4 (1.2)	13.9 (2.9)	10.7 (5.2)	<.001
	Slovenia	9.2 (3.0)	10.4 (2.2)	6.2 (2.1)	<.001
	EEB	18.2 (2.5)	12.5 (2.3)	14.5 (5.6)	<.001
	Other	20.3 (3.8)	18.5 (2.6)	11.4 (5.6)	<.001
	Age groups (%)				
	0–6	6.1 (1.1)	6.4 (0.6)	5.4 (0.9)	.006
	7–14	9.8 (1.5)	12.1 (1.4)	10.2 (1.8)	<.001
	15–24	8.1 (1.5)	10.5 (1.6)	7.5 (1.4)	<.001
	25–60	62.0 (2.6)	61.4 (1.6)	60.3 (3.6)	.012
	61–100	14.1 (3.9)	9.7 (1.7)	16.6 (3.9)	<.001
Dinner	Number of guests	355.8 (33.6) [0.2; −1.1]	399.7 (27.2) [−0.1; −0.8]	351.9 (56.7) [−1.4; 1.4]	.001
	First day (%)	23.8 (7.3)	22.1 (7.4)	25.0 (8.3)	.022
	Last day (%)	22.7 (8.2)	22.9 (8.1)	25.2 (11.6)	<.001
	Male (%)	47.7 (2.0)	47.5 (1.1)	48.3 (1.8)	.726
	Country of origin (%)				
	Austria	18.2 (3.6)	20.3 (3.5)	22.2 (7.7)	<.001
	Italy	9.5 (4.3)	9.8 (4.0)	23.8 (9.2)	<.001
	Germany	13.7 (2.6)	16.2 (3.1)	13.1 (3.9)	<.001
	Hungary	11.5 (2.6)	14.1 (3.4)	10.7 (5.8)	<.001
	Slovenia	11.4 (4.2)	11.5 (2.3)	6.5 (2.5)	<.001
	EEB	17.4 (2.6)	10.7 (2.5)	14.8 (6.8)	<.001
	Other	18.2 (4.0)	17.3 (3.6)	8.9 (4.8)	<.001
	Age groups (%)				
	0–6	4.2 (1.0)	4.1 (0.9)	3.7 (0.7)	.295
	7–14	11.2 (1.3)	13.0 (2.2)	10.9 (1.6)	<.001
	15–24	8.1 (1.5)	10.5 (1.9)	7.5 (1.7)	<.001
	25–60	62.8 (3.6)	62.7 (2.1)	61.9 (3.3)	.358
	61–100	13.7 (4.4)	9.8 (2.0)	15.9 (4.2)	<.001

*Note*. For the number of guests in square brackets also the skewness (first) and the excess kurtosis (second) are given. The remaining Eastern European countries and countries on the Balkans are combined in one category abbreviated as EEB. *p*-values are reported for likelihood ratio tests assessing differences between experimental conditions separately for each meal and variable/category while controlling for weekday.

Estimating average plate waste while controlling for the proportion of first day guests, last day guests, guests from different countries of origin, and guests of different age groups, confirms that neither of the two experimental conditions providing information to guests and asking them explicitly to reduce the plate waste they generate, significantly reduce the amount of plate waste generated (breakfast: *F* = 0.23, *p* = .79; dinner: *F* = 0.23, *p* = .79). These results are complemented by the mean and 95% confidence intervals for the effect estimates of difference in average plate waste for each experimental condition compared to the control group given in Table 4.

**Table 4.** Mean and 95% Confidence Intervals (Indicated by Their Lower and Upper Bound) of the Effect of the Experimental Condition Compared to the Control Group on Average Plate Waste per Person (in g) for Each Meal Estimated Using Weighted Linear Regression Models With Guest Characteristics as Control Variables.

Experimental condition	Breakfast			Dinner		
	Mean	Lower bound	Upper bound	Mean	Lower bound	Upper bound
Environmental beliefs	−1.56	−6.67	3.54	0.52	−10.52	11.56
Humor	0.67	−6.59	7.94	7.37	−14.57	29.32

The results from this quasi-experimental field study indicate that neither of the two messages designed to entice tourists to eat up everything they have taken from buffet onto their plate are effective in reducing the food waste these tourists generate. This conclusion stands in direct contradiction to the findings from the survey experiment, where the environmental belief intervention showed promising results with a significant increase in behavioral intentions to eat up all the food on the plate. There are a few plausible explanations for this phenomenon. First, it is possible that contextual or situational variables (Stern, 2000) prevented tourists from translating their intentions into behavior. Although we know from the survey study that the environmental belief message was successful in changing people’s beliefs and in altering behavioral intentions, other causes, including unfamiliarity with foods on offer, fear of missing out, laziness, and unconscious overserving, may still lead to people wasting food at buffets (Dolnicar & Juvan, 2019). Second, survey respondents may have been influenced by social desirability bias, which would make them respond in a more socially acceptable way (R. J. Fisher, 1993; Paulhus, 1984). This is a plausible explanation given that eating up what is on one’s plate represents a socially desirable behavior in many cultures (Liu et al., 2022). Third, the humor-based message was based on feline obesity. About a quarter of cat owners hold a positive attitude toward obese cats and associate chubbiness and fatness with cuteness (Churchill & Ward, 2016; Teng et al., 2020). Consequently, the lack of problem awareness among some tourists may have undermined the effectiveness of the humorous message. This explanation is further supported by the survey results from study 1, where participants found the humorous message more entertaining and amusing, but less concerning and guilt-inducing compared to the environmental belief message.

Although both messages failed to translate behavioral intentions into real behavior, it is noteworthy that the humor-based message caused fewer negative emotions, such as being upset or annoyed, than the environmental belief message. The humor-based message, in fact, triggered more positive emotions, such as being entertained or amused, than the environmental belief message. With vacations representing a time of enjoyment and entertainment (E. Cohen, 2011), the humor message shows better alignment (Prebensen et al., 2014); yet it did not reduce plate waste in the survey and field experiment.

## Conclusions

The aim of this study was to determine whether survey experiments can provide accurate estimates about the effectiveness of behavior change interventions relating to environmentally sustainable behaviors in real tourism settings. Based on the comparison of plate waste reduction derived from a survey experiment and a quasi-experimental field study, we conclude that this is not the case. In the survey experiment, tourists were over-reporting their intention not to generate plate waste, leading to the incorrect conclusion that the environmental belief message has the potential to significantly reduce plate waste at real hotel buffets.

This finding is not limited to the context of plate waste reduction. Rather, it has immediate implications for tourism researchers working on developing and empirically testing the effectiveness of behavior change interventions for sustainable tourism more broadly. The results of our study further demonstrate that, compared to self-report survey studies, field experiments are a more effective tool for drawing valid conclusions, as they can overcome the attitude-behavior gap. Researchers, therefore, should consider employing field experiments whenever possible. This is particularly important when research aims to derive tangible managerial recommendations in terms of operational changes that can be implemented to entice tourists to behave in more environmentally sustainable ways, newly developed behavior change interventions must be tested in field studies to be able to conclude with certainty that they indeed have a significant impact on tourist behavior.

Our study also has several limitations. First, the present study has been conducted only in the context of plate waste and has tested only two specific messages. It is possible that results would be slightly different for environmentally sustainable behaviors less affected by social desirability bias. Second, the timing for data collection differs between the survey experiment and the quasi-experimental field study. There is a 2-year gap between the field study and the survey experiment due to the disruption caused by the COVID-19 pandemic. However, as predicted during COVID, the tourism industry has returned to business as usual (Zhu & Dolnicar, 2022) and the hotel where the field study was conducted has resumed normal pre-COVID operations. Third, because of the nature of field experimentation, it is impossible to control the sample characteristics of the hotel guests that were at the hotel during the field experiment. To minimize the potential impact of this limitation, we have set strict filters for recruiting survey respondents to ensure a similar segment of the travel market was captured.

Finally, we share lessons we have learned from running a quasi-experimental field study to test the validity of survey experiments for determining the effectiveness of interventions aimed at making tourists behave in more sustainable ways. First, field studies cannot be implemented without the full cooperation of a committed industry partner. Full cooperation typically means buy-in from top management, which ensures that all staff are informed about the study and contribute, if necessary. Because of the need for industry collaboration and buy-in, it is difficult to conduct field studies using unrealistic interventions—interventions managers would never implement, even if they were to be proven effective in research. We acknowledge that this may stand in the way of perfect scientific designs, but, when the focus is on impact, there indeed is no point testing interventions that would not be adopted by industry. Another observation is that field studies cannot be implemented quickly. They take a lot of time and effort. As such, in today’s quantity-focused academic environment, they probably represent a less attractive research method, compared to online surveys or the use of big data. Yet, if the focus is on being able to draw valid conclusions about how changes affect any kind of actual behavior, field studies are unavoidable (Dolnicar, Gray et al., 2023), especially if researchers aspire to being able to provide reliable advice to industry about what they can do to change tourist behavior.

## Supplemental Material

sj-docx-1-jtr-10.1177_00472875241253009 – Supplemental material for On the Importance of Field Studies for Testing Theory-Driven Behavioral Change Interventions in (Sustainable) Tourism Supplemental material, sj-docx-1-jtr-10.1177_00472875241253009 for On the Importance of Field Studies for Testing Theory-Driven Behavioral Change Interventions in (Sustainable) Tourism by Emil Juvan, Oscar Yuheng Zhu, Bettina Grün and Sara Dolnicar in Journal of Travel Research
