# Supplementary material for: On the Importance of Field Studies for Testing Theory-Driven Behavioral Change Interventions in (Sustainable) Tourism
Source: J Travel Res. 2024 May 31;64(6):1449–63. doi: 10.1177/00472875241253009 (PMC12121902; doi:10.1177/00472875241253009)
Supplement: sj-docx-1-jtr-10.1177_00472875241253009 – Supplemental material for On the Importance of Field Studies for Testing Theory-Driven Behavioral Change Interventions in (Sustainable) Tourism [file sj-docx-1-jtr-10.1177_00472875241253009.docx]

**Survey 1 (Control)**

PAGE 1

Have you stayed at a hotel in the past 5 years?

Yes

No

Have you eaten at a hotel buffet in the past 5 years?

Yes

No

PAGE 2

Please imagine that you are on a relaxing beach holiday. Your hotel booking includes dinner at an all-you-can-eat buffet. For most people buffets are a wonderful experience where they can try a range of different foods they usually would not be able to sample. As a result, sometimes, they leave uneaten food behind because they end up not enjoying certain foods, they don’t like combinations of foods they have taken or because they have overestimated how hungry they are. In fact, at dinner buffets in four-star hotels, the average guest leaves behind on their plate at the end of the meal about 100g of uneaten food – that’s the equivalent of three large doughnuts. Leaving food behind uneaten at the buffet is quite common.

PAGE 3

Now please go back to imagining your relaxing beach holiday.

Imagine you are at the dining room where the hotel is serving the all-you-can-eat dinner buffet.

How much of the food you take from the buffet would you leave behind uneaten?

Please give us your most realistic estimate.

I would leave behind …..% of the food I have taken from the buffet.

**Survey 2 (2 Experimental Conditions)**

PAGE 1

Have you stayed at a hotel in the past 5 years?

Yes

No

Have you eaten at a hotel buffet in the past 5 years?

Yes

No

PAGE 2

Please imagine that you are on a relaxing beach holiday. Your hotel booking includes dinner at an all-you-can-eat buffet. For most people buffets are a wonderful experience where they can try a range of different foods they usually would not be able to sample. As a result, sometimes, they leave uneaten food behind because they end up not enjoying certain foods, they don’t like combinations of foods they have taken or because they have overestimated how hungry they are. In fact, at dinner buffets in four-star hotels, the average guest leaves behind on their plate at the end of the meal about 100g of uneaten food – that’s the equivalent of three large doughnuts. Leaving food behind uneaten at the buffet is quite common.

PAGE 3

Now please go back to imagining your relaxing beach holiday.

Imagine you are at the dining room where the hotel is serving the all-you-can-eat dinner buffet.

On the table you see the following sign:

| Experimental condition 1 | Experimental condition 2 |
| --- | --- |
| 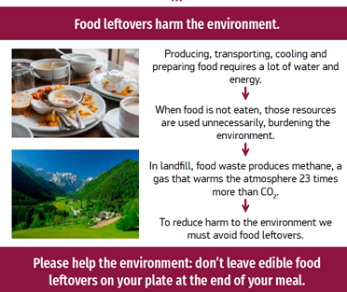 | 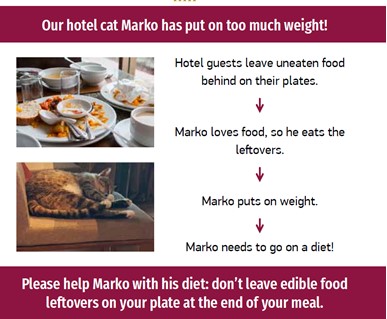 |

PAGE 4

How much of the food you take from the buffet would you leave behind uneaten?

Please give us your most realistic estimate.

I would leave behind …..% of the food I have taken from the buffet.

PAGE 5

Now please remember the sign you saw. How did it make you feel?

Annoyed  YES  NO

Wanting to eat up everything on my plate  YES  NO

Entertained  YES  NO

Guilty  YES  NO

Sustainable  YES  NO

Concerned  YES  NO

Upset  YES  NO

Responsible  YES  NO

Amused  YES  NO

Interested  YES  NO
